# Quantumness and Dequantumness Power of Quantum Channels

**DOI:** 10.3390/e24081146

**Published:** 2022-08-18

**Authors:** Hongting Song, Nan Li

**Affiliations:** 1 Qian Xuesen Laboratory of Space Technology, China Academy of Space Technology, Beijing 100094, China; 2 Academy of Mathematics and Systems Science, Chinese Academy of Sciences, Beijing 100190, China; 3 School of Mathematical Sciences, University of the Chinese Academy of Sciences, Beijing 100049, China

**Keywords:** quantumness in ensembles, quantum channel, quantum Markovianity, non-commutativity

## Abstract

Focusing on the dynamics of quantumness in ensembles, we propose a framework to qualitatively and quantitatively characterize quantum channels from the perspective of the amount of quantumness in ensembles that a quantum channel can induce or reduce. Along this line, the quantumness power and dequantumness power are introduced. In particular, once a quantum dynamics described by time-varying quantum channels reduces the quantumness for any input ensembles all the time, we call it a completely dequantumness channel, whose relationship with Markovianity is analyzed through several examples.

## 1. Introduction

As natural generalizations of transition matrices in stochastic analysis, quantum channels are completely positive and trace-preserving maps. A quantum channel usually changes the quantum features of the system, such as causing the decoherence of quantum states [1,2] and destroying the quantum correlations [3,4,5,6]. Characterizing quantum channels from the information perspective has received fruitful results. The entangling power [7], decorrelating capability [8], cohering and decohering power [9,10,11,12,13,14], and quantumness-generating capability [15] of quantum channels have been studied.

In this work, we propose a framework to qualitatively and quantitatively characterize quantum channels by analyzing the dynamics of quantumness in ensembles. A quantum ensemble $ℰ\;=\;\{\left(p_{i},\rho_{i}\right),i\in I\}$ is represented by a family of quantum states together with a probability distribution specifying the probability of the occurrence of each state [16]. It arises naturally in quantum mechanics and statistical physics, and is a fundamental and practical object in quantum information, especially in quantum measurement and quantum communication [17,18,19,20,21,22,23]. As long as the involved quantum states are not commutative, the quantum ensemble possesses a certain intrinsic quantum feature, which is named as quantumness in quantum ensembles. It plays a central role in quantum cryptography and other various quantum information processing tasks. Various measures of quantumness have been proposed from different perspectives, such as that via commutator [24,25], that based on no cloning and no broadcasting [19], that defined from the perspective of accessible information [24], and that via relative entropy [26] and coherence [27,28].

In general, the quantumness in a quantum ensemble will change after performing a quantum channel. It is natural to investigate the maximal amount of quantumness that a quantum channel can introduce or reduce. In this work, by virtue of the quantumness measure based on commutators [24] that is easy to calculate, we study the characterization of quantum channels from the perspective of quantumness power and dequantumness power, which quantify the maximal amount of quantumness that a quantum channel can induce and reduce, respectively. Comparing with the result in Ref. [29] where the quantumness of the channel is defined as the minimum average quantum coherence of the state space after the dynamics, quantumness power defined here is the maximal amount of the non-commutativity between the states that can be generated after the channel. The properties and calculation process of quantumness power and dequantumness power have been analyzed. We call a quantum dynamics described by a quantum channel a completely dequantumness channel if it reduces the quantumness in ensembles all the time. Through several significant examples, the relationship between the completely dequantumness channel and quantum Markovian channel is analyzed. It is worth mentioning that although we mainly focus on the qubit channels, without loss of generality, the result can be directly extended to qudit cases.

The paper is organized as follows. In Section 2, we briefly review the measure of quantumness adopted in this work. Quantumness power and dequantumness power of the quantum channel with their modified versions are introduced in Section 3. We give the definition of the completely dequantumness channel and investigate its relationship with quantum Markovianity through several significant examples in Section 4. We conclude with a summary in Section 5.

## 2. Measure of Quantumness

Based on the direct connection between the quantumness of an ensemble and the non-commutativity among its constituent states, the quantumness of the quantum ensemble $ℰ\;=\;\{\left(p_{i},\rho_{i}\right),i\in I\}$ can be naturally quantified via the commutator as [24]

(1) $$Q\left(ℰ\right)\;=\;-\sum_{i,j}p_{i}p_{j}\mathrm{tr}{\left[\rho_{i},\rho_{j}\right]}^{2},$$

where $\left[\rho_{i},\rho_{j}\right]\;=\;\rho_{i}\rho_{j}\;-\;\rho_{j}\rho_{i}$ stands for the commutator, which is anti-Hermitian. This measure is easy to calculate. We remark that in Refs. [30,31] the authors also used the Hilbert–Schmidt norm of the commutators between two density operators to quantity the non-commutativity between these two density operators.

For the two-qubit case, by virtue of the Bloch representation of the state, the expression of Q(ℰ) for ensembles with only two ingredients such that $ℰ\;=\;\{\left(p,\rho_{1}\right),\left(1\;-\;p,\rho_{2}\right)\}$ can be further derived. Here p∈(0,1), $\rho_{i}\;=\;\frac{1}{2}\left(1\;+\;{\vec{r}}_{i}\cdot \vec{\sigma }\right),\;i\;=\;1,2$ with 1 the identity operator, ${\vec{r}}_{i}\;=\;\left(r_{ix},r_{iy},r_{iz}\right)$ the Bloch vector of the state $\rho_{i}$, and $\vec{\sigma }\;=\;\left(\sigma_{1},\sigma_{2},\sigma_{3}\right)$ the vector of the Pauli matrices. Then, it can be calculated that

$$Q\left(ℰ\right)\;=\;p\left(1\;-\;p\right)|{\vec{r}}_{1}\;\times \;{\vec{r}}_{2}{|}^{2}\;=\;p\left(1\;-\;p\right)\left(r_{1}^{2}r_{2}^{2}\;-\;{\left({\vec{r}}_{1}\cdot {\vec{r}}_{2}\right)}^{2}\right).$$

Here $r_{i}^{2}\;=\;{\left|{\vec{r}}_{i}\right|}^{2}$, × and · denote the outer and inner product of the vectors, respectively. The Bloch vector of state $\rho_{i}$ can be given as ${\vec{r}}_{i}\;=\;r_{i}\left(\sin\theta_{i}\cos\phi_{i},\sin\theta_{i}\sin\phi_{i},\cos\theta_{i}\right)$ with $r_{i}\in \left[0,1\right],\;\theta_{i}\in \left[0,\pi \right]$, and $\phi_{i}\in \left[0,2\pi \right)$, then

(2) $$Q\left(ℰ\right)\;=\;p\left(1\;-\;p\right)r_{1}^{2}r_{2}^{2}\left(n_{1}^{2}\;+\;n_{2}^{2}\;+\;n_{3}^{2}\right),$$

with

(3) $$\begin{array}{cc} n_{1}\;=\; & \sin\theta_{1}\sin\theta_{2}\sin\left(\phi_{1}\;-\;\phi_{2}\right),\\ n_{2}\;=\; & \sin\theta_{1}\cos\phi_{1}\cos\theta_{2}\;-\;\cos\theta_{1}\sin\theta_{2}\cos\phi_{2},\\ n_{3}\;=\; & \sin\theta_{1}\sin\phi_{1}\cos\theta_{2}\;-\;\cos\theta_{1}\sin\theta_{2}\sin\phi_{2}. \end{array}$$

Recently, a modified version of this measure is proposed in Ref. [15] as

$$Q^{{}^{\prime }}\left(ℰ\right)\;=\;-\sum_{i,j}\sqrt{p_{i}p_{j}}\mathrm{tr}{\left[\sqrt{\rho_{i}},\sqrt{\rho_{j}}\right]}^{2},$$

which is proved to bear some nice properties, such as the positivity, unitary invariance, subaddtivity, concavity under probability union, convexity under state decomposition, and increasing under fine graining.

For simplicity in calculation, we adopt the measure in Equation (2) in the following. It is worth mentioning that all the work derived here can be directly generalized to other measures.

## 3. Quantumness and Dequantumness Power

After a quantum channel Λ, which is a linear, trace-preserving completely positive map, the ensemble $ℰ\;=\;\{\left(p_{i},\rho_{i}\right),i\in I\}$ evolves to $\Lambda \left(ℰ\right)\;=\;\{\left(p_{i},\Lambda \left(\rho_{i}\right)\right),i\in I\}$. By analyzing the dynamics of quantumness in ensembles, we can characterize the quantumness power and dequantumness power of the quantum channel. To be specific, the quantumness power of a quantum channel is defined as the maximal amount of quantumness that it generates over all input ensembles ℰ. Its expression is given as

$$C\left(\Lambda \right)\;=\;\max_{ℰ}(Q(\Lambda (ℰ))\;-\;Q(ℰ)),$$

which quantifies the ability to induce quantumness. If we only focus on the initial commutative ensembles, we can get another definition of quantumness power which we denote as $C^{{}^{\prime }}$ with expression

$$C^{{}^{\prime }}\left(\Lambda \right)\;=\;\max_{\left\{ℰ:\;Q(ℰ)\;=\;0\right\}}Q\left(\Lambda \left(ℰ\right)\right).$$

Similarly, we can define the dequantumness power of a quantum channel as the maximal amount by which the quantumness of the ensemble is reduced when it passed through the channel, i.e.,

$$D\left(\Lambda \right)\;=\;\max_{ℰ}(Q(ℰ)\;-\;Q(\Lambda (ℰ))).$$

When we only consider the initial ensembles with the maximal quantumness, we can obtain a modified version

$$\begin{array}{cc} D^{{}^{\prime }}\left(\Lambda \right) & \;=\;\max_{\left\{ℰ:\;Q\left(ℰ\right)\;=\;Q_{\max}\right\}}(Q(ℰ)\;-\;Q(\Lambda (ℰ)))\\ \; & \;=\;Q_{\max}\;-\;\min_{\left\{ℰ:\;Q\left(ℰ\right)\;=\;Q_{\max}\right\}}Q\left(\Lambda \left(ℰ\right)\right). \end{array}$$

Here $Q_{\max}$ denotes the maximal amount of quantunness in a quantum ensemble for a given Hilbert space, which is dependent on the space dimension.

We remark that in [32], the quantumness of a quantum channel is defined as the maximal quantumness (non-commutativity) between the output states of the quantum channel for any two maximal-quantumness states, which can be formally expressed as

$$\max_{\left\{ℰ:\;Q\left(ℰ\right)\;=\;Q_{\max}\right\}}Q\left(\Lambda \left(ℰ\right)\right).$$

Note that another difference in the motivation is that we start from the quantumness in ensembles rather than any two quantum states.

From the definition, we can obtain the following properties.

(1) $C\left(\Lambda \right)\geq C^{{}^{\prime }}\left(\Lambda \right)$ and $D\left(\Lambda \right)\geq D^{{}^{\prime }}\left(\Lambda \right)$.

(2) $C^{{}^{\prime }}\left(\Lambda \right)\;=\;0$ is equivalent to that Λ is a commutativity preserving channel.

(3) C(U) = 0 and D(U) = 0, where *U* is the unitary operation.

(4) $C\left(\Lambda_{1}\circ \Lambda_{2}\right)\leq C\left(\Lambda_{1}\right)\;+\;C\left(\Lambda_{2}\right)$ and $D\left(\Lambda_{1}\circ \Lambda_{2}\right)\leq D\left(\Lambda_{1}\right)\;+\;D\left(\Lambda_{2}\right)$.

(5) $C_{2}\left(\Lambda \right)\leq C_{n}\left(\lambda \right)<2C_{2}\left(\Lambda \right)$ and $D_{2}\left(\Lambda \right)\leq D_{n}\left(\lambda \right)<2D_{2}\left(\Lambda \right)$, where $C_{n}\left(\Lambda \right)$ and $D_{n}\left(\Lambda \right)$ represent the quantumness and dequantumness power defined on the ensembles with less than *n* ingredients, respectively.

**Proof.**  Since the first four properties can be directly verified from the definition, we only prove the property (5) as follows. For simplicity, we just give the proof of the quantumness power, with the case of the dequantumness power similarly derived.

For ensembles having two ingredients ${ℰ}_{2}\;=\;\left\{\left(p,\rho_{1}\right),\left(1\;-\;p,\rho_{2}\right)\right\}$, the quantumness power is

$$\begin{array}{cc} C_{2}\left(\Lambda \right)\;=\; & \max_{{ℰ}_{2}}(Q(\Lambda (ℰ))\;-\;Q(ℰ))\;=\;\max_{p,\rho_{1},\rho_{2}}2p\left(1\;-\;p\right)\left(\mathrm{tr}{\left[\rho_{1},\rho_{2}\right]}^{2}\;-\;\mathrm{tr}{\left[\Lambda \left(\rho_{1}\right),\Lambda \left(\rho_{2}\right)\right]}^{2}\right)\\ \;=\; & \frac{1}{2}\max_{\rho_{1},\rho_{2}}\left(\mathrm{tr}{\left[\rho_{1},\rho_{2}\right]}^{2}\;-\;\mathrm{tr}{\left[\Lambda \left(\rho_{1}\right),\Lambda \left(\rho_{2}\right)\right]}^{2}\right)≜\frac{1}{2}H. \end{array}$$

For ensembles with less than *n* constitutes denoted by ${ℰ}_{n}$, the quantumness power is

$$\begin{array}{cc} C_{n}\left(\Lambda \right)\;= & \max_{\left\{{ℰ}_{2},\cdots ,{ℰ}_{n}\right\}}(Q\left(\Lambda \left({ℰ}_{n}\right)\right)\;-\;Q\left({ℰ}_{n}\right))\;=\;\max_{k\;=\;2,\cdots ,n}\max_{{ℰ}_{k}}(Q\left(\Lambda \left({ℰ}_{k}\right)\right)\;-\;Q\left({ℰ}_{k}\right))\\ \;= & \max_{k\;=\;2,\cdots ,n}\max_{\left\{\left(p_{i},\rho_{i}\right),i\;=\;1,\cdots ,k\right\}}\sum_{i\neq j}p_{i}p_{j}\left(\mathrm{tr}{\left[\rho_{i},\rho_{j}\right]}^{2}\;-\;\mathrm{tr}{\left[\Lambda \left(\rho_{i}\right),\Lambda \left(\rho_{j}\right)\right]}^{2}\right)\\ \;\leq & \max_{k\;=\;2,\cdots ,n}\max_{\left\{p_{i},i\;=\;1,\cdots ,k\right\}}\sum_{i\neq j}p_{i}p_{j}H\;=\;\max_{k\;=\;2,\cdots ,n}\max_{\left\{p_{i},i\;=\;1,\cdots ,k\right\}}\left(1\;-\;\sum_{i}p_{i}^{2}\right)H\\ \;\leq & \max_{k\;=\;2,\cdots ,n}\left(1\;-\;\frac{1}{k}\right)H<2C_{2}\left(\Lambda \right), \end{array}$$

meanwhile $C_{n}\left(\Lambda \right)\geq C_{2}\left(\Lambda \right)$, then we can directly get that

$$C_{2}\left(\Lambda \right)\leq C_{n}\left(\Lambda \right)<2C_{2}\left(\Lambda \right),\;\;\;\;\;\;n\geq 2.$$

□

From this property, we can obtain that the calculation of quantumness power and dequantumness power can be restricted to the ensembles with two ingredients. In the following, focusing on one particular channel, the explicit calculation process is given.

**Example** **1.** *For amplitude damping channels the Kraus operators of which are $E_{0}\;=\;\left(\begin{array}{cc} 1 & 0\\ 0 & \sqrt{1\;-\;\lambda } \end{array}\right)$ and $E_{1}\;=\;\left(\begin{array}{cc} 0 & \sqrt{\lambda }\\ 0 & 0 \end{array}\right)$, we can calculate the quantumness power and dequantumness power as follows.*

Through this channel, the Bloch vectors of the states in the ensemble ${ℰ}_{2}\;=\;\left\{\left(p,\rho_{1}\right),\left(1\;-\;p,\rho_{2}\right)\right\}$ change from $r_{i}\left(\sin\theta_{i}\cos\phi_{i},\sin\theta_{i}\sin\phi_{i},\cos\theta_{i}\right)$ to

$$\begin{array}{c} {\vec{r}}_{i}\;=\;\left(r_{i}\sin\theta_{i}\cos\phi_{i}\sqrt{1\;-\;\lambda },r_{i}\sin\theta_{i}\sin\phi_{i}\sqrt{1\;-\;\lambda },\lambda \;+\;\left(1\;-\;\lambda \right)r_{i}\cos\theta_{i}\right). \end{array}$$

From Equation (2), we can obtain the quantumness of this evolved ensemble as

(4) $$\begin{array}{cc} Q(\Lambda \left({ℰ}_{2}\right))\;=\; & p\left(1\;-\;p\right)\left(1\;-\;\lambda \right)[\lambda^{2}\left(h_{1}^{2}\;+\;h_{2}^{2}\right)\;+\;r_{1}^{2}r_{2}^{2}\left(n_{2}^{2}\;+\;n_{3}^{2}\right)\\ \; & \;+\;r_{1}^{2}r_{2}^{2}\left(1\;-\;\lambda \right)n_{1}^{2}\;+\;2\lambda r_{1}r_{2}\left(n_{3}h_{1}\;+\;n_{2}h_{2}\right)], \end{array}$$

where $n_{i}$ are the same to the ones in Equation (3), and

$$\begin{array}{c} h_{1}\;=\;r_{1}\sin\theta_{1}\sin\phi_{1}\;-\;r_{2}\sin\theta_{2}\sin\phi_{2}\;+\;r_{1}r_{2}\left(\cos\theta_{1}\sin\theta_{2}\sin\phi_{2}\;-\;\sin\theta_{1}\cos\theta_{2}\sin\phi_{1}\right),\\ h_{2}\;=\;r_{1}\sin\theta_{1}\cos\phi_{1}\;-\;r_{2}\sin\theta_{2}\cos\phi_{2}\;+\;r_{1}r_{2}\left(\cos\theta_{1}\sin\theta_{2}\cos\phi_{2}\;-\;\sin\theta_{1}\cos\theta_{2}\cos\phi_{1}\right). \end{array}$$

The quantumness power restricted to the ensembles with two ingredients is

$$C_{2}\left(\Lambda \right)\;=\;\max_{p,r_{i},\theta_{i},\phi_{i}}(Q\left(\Lambda \left({ℰ}_{2}\right)\right)\;-\;p\left(1\;-\;p\right)r_{1}^{2}r_{2}^{2}\left(n_{1}^{2}\;+\;n_{2}^{2}\;+\;n_{3}^{2}\right)).$$

**Figure 1:**
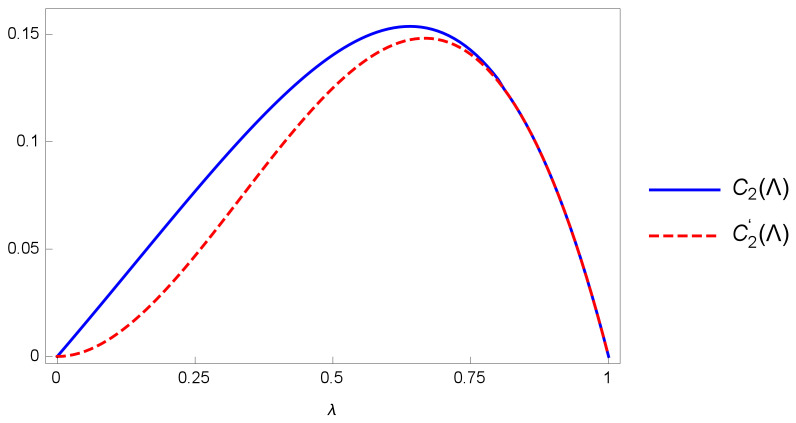
The graphs of $C_{2}$ and $C_{2}^{{}^{\prime }}$ for the amplitude damping channel.

Since the optimization is very complicated, we only show the numerical result as the blue solid line in Figure 1.

If we only focus on the initial ensembles without quantumness, i.e., $Q({ℰ}_{2})\;=\;0$, which means $r_{1}$ (or $r_{2})\;=\;0$ or $n_{1}\;=\;n_{2}\;=\;n_{3}\;=\;0$, we can get the expression of the modified quantumness power as

(5) $$C_{2}^{{}^{\prime }}\left(\Lambda \right)\;=\;\lambda^{2}\left(1\;-\;\lambda \right),$$

whose proof is left in the Appendix A. The difference between these two measures is shown in Figure 1. $C_{2}\left(\Lambda \right)>C_{2}^{{}^{\prime }}\left(\Lambda \right)$ when $0\leq \lambda <\lambda_{c}$ and $C_{2}\left(\Lambda \right)\;=\;C_{2}^{{}^{\prime }}\left(\Lambda \right)$ when $\lambda_{c}<\lambda \leq 1$, where $\lambda_{c}\approx 0.75$.

Similarly, we can get the expression of dequantumness power as

$$D_{2}\left(\Lambda \right)\;=\;\max_{p,r_{i},\theta_{i},\phi_{i}}(p\left(1\;-\;p\right)r_{1}^{2}r_{2}^{2}\left(n_{1}^{2}\;+\;n_{2}^{2}\;+\;n_{3}^{2}\right)\;-\;Q\left(\Lambda \left({ℰ}_{2}\right)\right)).$$

Noting $\max_{p,r_{i},\theta_{i},\phi_{i}}Q\left({ℰ}_{2}\right)\;=\;\frac{1}{4}$ with $p\;=\;\frac{1}{2},\;r_{1}\;=\;r_{2}\;=\;1$ and $\sin\theta_{1}\sin\theta_{2}\cos\left(\phi_{1}\;-\;\phi_{2}\right)\;=\;-\cos\theta_{1}\cos\theta_{2}$, the modified dequantumness power is

$$\begin{array}{cc} D_{2}^{{}^{\prime }}\left(\Lambda \right)\;=\; & \max_{\theta \in [0,\pi ]}\frac{1}{4}[1\;-\;\left(1\;-\;\lambda \right)[(2\lambda^{2}\;-\;2\lambda \;+\;1)\\ \; & \pm 2\lambda^{2}\sin\theta \cos\theta \;+\;2\lambda \left(1\;-\;\lambda \right)(\cos\theta \pm \sin\theta )]]. \end{array}$$

**Figure 2:**
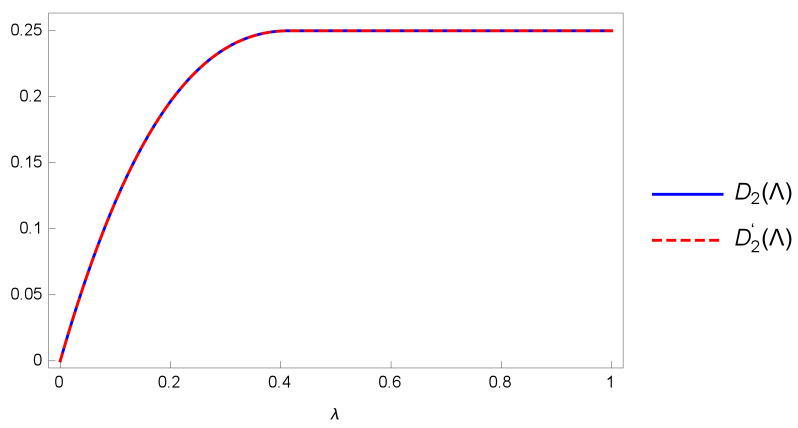
The graphs of $D_{2}$ and $D_{2}^{{}^{\prime }}$ for the amplitude damping channel.

As shown in Figure 2, $D_{2}\left(\Lambda \right)\;=\;D_{2}^{{}^{\prime }}\left(\Lambda \right)$ in this case.

From this example, we can obtain that C(Λ) can be strictly larger than $C^{{}^{\prime }}\left(\Lambda \right)$, which means that the maximum may not be achieved at the free case just like the cohering power [14]. Though $D_{2}\left(\Lambda \right)\;=\;D_{2}^{{}^{\prime }}\left(\Lambda \right)$ in this case, we conjecture this equality may fail in certain cases. But we have not found the counterexample satisfying $D_{2}\left(\Lambda \right)>D_{2}^{{}^{\prime }}\left(\Lambda \right)$ yet.

Meanwhile, we can obtain that the channel with higher quantumness power does not necessarily have stronger or weaker dequantumness power. The relationship among them is complicated. For example, $C_{2}\left(0.25\right)>C_{2}\left(0.99\right)$ while $D_{2}\left(0.25\right)<D_{2}\left(0.99\right)$, $C_{2}\left(0.5\right)>C_{2}\left(0.1\right)$ and $D_{2}\left(0.5\right)>D_{2}\left(0.1\right)$.

## 4. Completely Dequantumness Channel and Its Relationship with Quantum Markovianity

In this section, we consider a quantum channel as a quantum evolution $\Lambda_{t}$. If for all the quantum ensembles, the channel reduces the quantumness all the time, we call this channel as the completely dequantumness channel. For these channels, we always have

$$\frac{d}{dt}Q\left(\Lambda_{t}\left(ℰ\right)\right)\leq 0,\;\;\;\forall ℰ,\;\forall t\geq 0.$$

The completely dequantumness channel can be verified to satisfy the following properties:

(1) The quantumness power of the completely dequantumness channel is always 0, while the inverse is not always true.

(2) To verify whether a channel is a completely dequantumness channel or not, we only need to verify whether the inequality $\frac{d}{dt}Q\left(\Lambda_{t}\left(ℰ\right)\right)\leq 0$ holds or not for all the ensembles with two ingredients.

**Proof.**  We only give the proof of property (2) since the first one can be verified directly from the definition. If the channel reduces quantumness for all ensembles $ℰ\;=\;\{\left(p_{i},\rho_{i}\right),i\in I\}$, we can directly obtain that for the ensembles with two ingredients $ℰ\;=\;\{\left(p,\rho_{1}\right),\left(1\;-\;p,\rho_{2}\right)\}$, the inequality $\frac{d}{dt}Q\left(\Lambda_{t}\left(ℰ\right)\right)\leq 0$ holds.

Conversely, if for all the ensembles with two ingredients, the inequality holds, then by virtue of the definition in Equation (1), for the general ensembles with arbitrary numbers of ingredients, we can obtain that $\frac{d}{dt}Q\left(\Lambda_{t}\left(ℰ\right)\right)\;=\;-\sum_{i,j\in I}p_{i}p_{j}\frac{d}{dt}\mathrm{tr}{\left[\Lambda_{t}\left(\rho_{i}\right),\Lambda_{t}\left(\rho_{j}\right)\right]}^{2}\leq 0$. □

For open quantum systems, the definition of completely dequantumness channel (dynamics) reflects the information flow of quantumness from the quantum system to the environment. Since the information loss is a typical feature of Markovianity, it is natural to investigate the relationship between the completely-dequantumness property of a quantum dymamics and its Markovianity.

It is worth mentioning that there are various criteria proposed to qualitatively or quantitatively characterize quantum non-Markovianity from different perspectives, such as divisibility [33,34,35,36], the distinguishability of states [37,38], fidelity [39], correlations [35,40,41], Fisher information [42,43,44], and Rényi entropy [45]. Among these, a criterion that can fully characterize the non-Markovianity of a quantum dynamics [33] is using the appearance of negative decoherence rates in the canonical form of the master equation

$$\frac{d\rho_{t}}{dt}\;=\;-\frac{i}{\hbar }\left[H\left(t\right),\rho_{t}\right]\;+\;\sum_{k\;=\;1}^{d^{2}\;-\;1}\gamma_{k}\left(t\right)[L_{k}\left(t\right)\rho_{t}L_{k}{\left(t\right)}^{†}\;-\;\frac{1}{2}\left\{L_{k}{\left(t\right)}^{†}L_{k}\left(t\right),\rho_{t}\right\}],$$

where the $L_{k}\left(t\right)$ form an orthonormal basis set of traceless operators, i.e., $\mathrm{tr}L_{k}\left(t\right)\;=\;0,$$\mathrm{tr}L_{j}\left(t\right)L_{k}{\left(t\right)}^{†}\;=\;\delta_{jk},$ and H(t) is Hermitian. In this sense, a time-local master equation is Markovian if and only if the canonical decoherence rates are positive at any time, i.e.,

(6) $$\gamma_{k}\left(t\right)\geq 0,\;\forall t\geq 0,\;k\;=\;1,\cdots ,d^{2}\;-\;1.$$

More importantly, the authors in Ref. [33] give an example of a master equation that is non-Markovian for all times t≥0, but to which nearly all proposed non-Markovian measures do not work. For this reason, we will adopt this criterion for Markovianity.

To make a comparative study between the completely-dequantumness property and the Markovianity, we focus on phase damping dynamics, amplitude damping dynamics, and random unitary dynamics.

### 4.1. Phase Damping Dynamics

Consider the qubit dynamics $\Lambda \;=\;\{\Lambda_{t}:t\geq 0\}$ with $\rho_{t}\;=\;\Lambda_{t}\left(\rho \right)$ described by the differential equation

$$\frac{d\rho_{t}}{dt}\;=\;\gamma_{t}\left(\sigma_{z}\rho_{t}\sigma_{z}\;-\;\rho_{t}\right),$$

where $\int_{0}^{t}\gamma_{s}ds\geq 0$ and $\sigma_{z}$ is the Pauli-*z* spin matrix.

This dynamics is actually a phase damping channel and can be presented as $\Lambda_{t}\left(\rho \right)\;=\;E_{0}\rho E_{0}^{†}\;+\;E_{1}\rho E_{1}^{†}$ with Kraus operators $E_{0}\;=\;\mathrm{diag}\left(1,\sqrt{1\;-\;\lambda_{t}}\right)$ and $E_{1}\;=\;\mathrm{diag}\left(0,\sqrt{\lambda_{t}}\right)$, where $\lambda_{t}\;=\;1\;-\;e^{-4\int_{0}^{t}\gamma_{s}ds}$.

The Bloch vectors of the evolved states are

$${\vec{r}}_{i}\left(t\right)\;=\;r_{i}\left(\sqrt{1\;-\;\lambda_{t}}\sin\theta_{i}\cos\phi_{i},\sqrt{1\;-\;\lambda_{t}}\sin\theta_{i}\sin\phi_{i},\cos\theta_{i}\right).$$

The quantumness of evolved ensemble $\Lambda_{t}\left(ℰ\right)\;=\;\left\{\left(p,\Lambda_{t}\left(\rho_{1}\right)\right),\left(1\;-\;p,\Lambda_{t}\left(\rho_{2}\right)\right)\right\}$ turns out to be

$$Q\left(\Lambda_{t}\left(ℰ\right)\right)\;=\;p\left(1\;-\;p\right)r_{1}^{2}r_{2}^{2}\left[{\left(1\;-\;\lambda_{t}\right)}^{2}m_{1}\;+\;\left(1\;-\;\lambda_{t}\right)\left(m_{2}\;+\;m_{3}\right)\right],$$

with $m_{i}\;=\;n_{i}^{2}$ given in Equation (3), and the derivative is

$$\begin{array}{cc} \frac{dQ(\Lambda_{t}\left(ℰ\right))}{dt}\;=\; & -p\left(1\;-\;p\right)r_{1}^{2}r_{2}^{2}[2\left(1\;-\;\lambda_{t}\right)m_{1}\;+\;m_{2}\;+\;m_{3}]\frac{d\lambda_{t}}{dt}\propto \;-\;\gamma_{t}. \end{array}$$

From above, we can obtain that for all quantum ensembles,

$$\frac{dQ(\Lambda_{t}\left(ℰ\right))}{dt}\leq 0\;\mathrm{if}\;\mathrm{and}\;\mathrm{only}\;\mathrm{if}\;\gamma_{t}\geq 0.$$

It can be directly verified from the definition of Equation (6) that $\gamma_{t}\geq 0$ is just the condition that the channel $\Lambda_{t}$ is Markovian, which is also in accordance with the results revealed by the measures based on the quantum trace distance (BLP-Markovianity) [37], dynamical divisibility (RHP-Markovianity) [35], quantum mutual information (LFS-Markovianity) [41], and quantum Fisher information [43] (see Refs. [41,43] and references therein). This implies that for the phase damping dynamics, it is completely dequantumness if and only if it is Markovian.

### 4.2. Amplitude Damping Dynamics

Consider the qubit dynamics $\Lambda \;=\;\{\Lambda_{t}:t\geq 0\}$ with $\rho_{t}\;=\;\Lambda_{t}\left(\rho \right)$ satisfying the following master equation

$$\frac{d\rho_{t}}{dt}\;=\;-\frac{i}{2}s_{t}\left[\sigma_{+}\sigma_{-},\rho_{t}\right]\;+\;\gamma_{t}\left(\sigma_{-}\rho_{t}\sigma_{+}\;-\;\frac{1}{2}\left\{\sigma_{+}\sigma_{-},\rho_{t}\right\}\right),$$

where {·,·} denotes the anti-commutator, $\sigma_{\pm }$ are the atomic raising and lowing operators, respectively, and $s_{t}\;=\;-2ℑ\frac{{\dot{G}}_{t}}{G_{t}}$, $\gamma_{t}\;=\;-2ℜ\frac{{\dot{G}}_{t}}{G_{t}}$. Here $G_{t}$ satisfies the equation ${\dot{G}}_{t}\;=\;\;-\;\int_{0}^{t}f_{t-s}G_{s}ds$ with initial condition $G_{0}\;=\;1$, and $f_{t}$ is the reservoir correlation function.

This dynamics is actually an amplitude damping channel. We can directly obtain the Bloch vectors of the evolved states in ensemble $\Lambda_{t}\left(ℰ\right)$ as

$$\begin{array}{cc} {\vec{r}}_{i}\left(t\right)\;=\; & \left(r_{i}|G_{t}|\sin\theta_{i}\cos\left(\phi_{i}\;+\;\delta_{t}\right),r_{i}|G_{t}|\sin\theta_{i}\sin\left(\phi_{i}\;+\;\delta_{t}\right),1\;-\;{\left|G_{t}\right|}^{2}\left(1\;-\;r_{i}\cos\theta_{i}\right)\right). \end{array}$$

Here $\delta_{t}$ is the argument of $G_{t}$. The derivative of quantumness of this evolved ensemble can be calculated as

$$\begin{array}{cc} \frac{dQ(\Lambda_{t}\left(ℰ\right))}{d|G_{t}{|}^{2}}\;=\; & p\left(1\;-\;p\right)[k_{12}\left(t\right)f_{12}\left(t\right)\;+\;2{\left|G_{t}\right|}^{2}r_{1}^{2}r_{2}^{2}\sin^{2}\theta_{1}\sin^{2}\theta_{2}\sin^{2}\left(\phi_{1}\;-\;\phi_{2}\right)\\ \; & \;+k_{21}\left(t\right)f_{21}\left(t\right)\;-\;2r_{1}r_{2}\sin\theta_{1}\sin\theta_{2}\cos\left(\phi_{1}\;-\;\phi_{2}\right)l(t)], \end{array}$$

where

$$\begin{array}{cc} k_{ij}\left(t\right)\;=\; & r_{i}\sin\theta_{i}\left(1\;-\;\right|G_{t}{|}^{2}\left(1\;-\;r_{j}\cos\theta_{j}\right)),\;\;\;i,j\;=\;1,2,\\ f_{ij}\left(t\right)\;=\; & r_{i}\sin\theta_{i}\left(1\;-\;3\right|G_{t}{|}^{2}\left(1\;-\;r_{j}\cos\theta_{j}\right)),\;\;\;i,j\;=\;1,2,\\ l(t)\;=\; & 1\;-\;2|G_{t}{|}^{2}\left(2\;-\;r_{1}\cos\theta_{1}\;-\;r_{2}\cos\theta_{2}\right)\;+\;3{\left|G_{t}\right|}^{4}\left(1\;-\;r_{1}\cos\theta_{1}\right)\left(1\;-\;r_{2}\cos\theta_{2}\right). \end{array}$$

**Figure 3:**
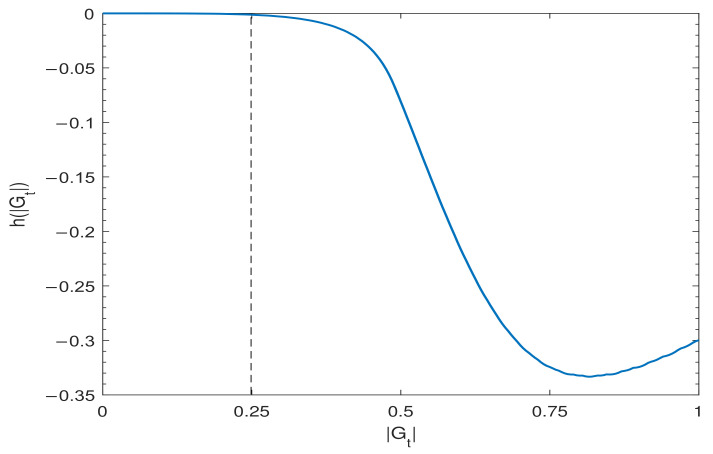
$h(|G_{t}|)$ as a function of $|G_{t}|$.

We define

$$h(|G_{t}\left|\right)≜\min_{p,r_{i},\theta_{i},\phi_{i}}\frac{dQ(\Lambda_{t}\left(ℰ\right))}{d|G_{t}{|}^{2}}$$

and plot it in Figure 3. From the figure, we can easily get that $h(|G_{t}|)<0$ when $|G_{t}|>\frac{1}{4}$, which implies that

$$\frac{dQ(\Lambda_{t}\left(ℰ\right))}{dt}\leq 0,\;\forall \;ℰ\;\;\Leftrightarrow \;\;\left|G_{t}\right|\leq \frac{1}{4}\;\mathrm{and}\;\frac{d|G_{t}|}{dt}\leq 0.$$

If $|G_{t}|>\frac{1}{4}$, we can always find particular ensemble whose quantumness increases during the evolution.

It can be directly verified from the definition of Equation (6) that the amplitude damping channel is Markovian if and only if $\gamma_{t}\;=\;\;-\;\frac{2}{|G_{t}|}\frac{d|G_{t}|}{dt}\geq 0$, i.e., $\frac{d|G_{t}|}{dt}\leq 0$, which is also in accordance with the result revealed by the measures based on the quantum trace distance (BLP-Markovianity), quantum mutual information (LFS-Markovianity), and quantum Fisher information (see Refs. [41,43,46] and references therein). Based on this observation, we know that Markovianity does not imply completely dequantumness. It means that there exists a Markovian channel that can induce quantumness for some ensembles.

### 4.3. Random Unitary Dynamics

Consider the qubit dynamics $\Lambda \;=\;\{\Lambda_{t}:t\geq 0\}$ with $\rho_{t}\;=\;\Lambda_{t}\left(\rho \right)$ described by the master equation

$$\frac{d\rho_{t}}{dt}\;=\;\sum_{i\;=\;1}^{3}\gamma_{i}\left(t\right)\left(\sigma_{i}\rho_{t}\sigma_{i}\;-\;\rho_{t}\right),$$

where $\gamma_{i}\left(t\right)$ are suitable real functions of time, and $\sigma_{i}$ are the Pauli spin matrices. This dynamic is actually a random unitary dynamic and can be written in the following equivalent form

$$\Lambda_{t}\left(\rho \right)\;=\;\sum_{i\;=\;0}^{3}p_{i}\left(t\right)\sigma_{i}\rho \sigma_{i}.$$

Here $p_{0}\left(t\right)\;=\;\left(1\;+\;\sum_{j\;=\;1}^{3}\lambda_{j}\left(t\right)\right)/4$ and $p_{i}\left(t\right)\;=\;\lambda_{i}\left(t\right)/2\;+\;\left(1\;-\;\sum_{j\;=\;1}^{3}\lambda_{j}\left(t\right)\right)/4$ with $\lambda_{i}\left(t\right)\;=\;e^{2\int_{0}^{t}\left(\gamma_{i}\left(s\right)\;-\;\sum_{j\;=\;1}^{3}\gamma_{j}\left(s\right)\right)ds}$.

The Bloch vectors of the evolved ensemble $\Lambda_{t}\left(ℰ\right)$ can be derived as

$${\vec{r}}_{i}\left(t\right)\;=\;r_{i}\left(\lambda_{1}\left(t\right)\sin\theta_{i}\cos\phi_{i},\lambda_{2}\left(t\right)\sin\theta_{i}\sin\phi_{i},\lambda_{3}\left(t\right)\cos\theta_{i}\right),$$

and the quantumness measure is

$$Q\left(\Lambda_{t}\left(ℰ\right)\right)\propto \lambda_{1}^{2}\left(t\right)\lambda_{2}^{2}\left(t\right)m_{1}\;+\;\lambda_{1}^{2}\left(t\right)\lambda_{3}^{2}\left(t\right)m_{2}\;+\;\lambda_{2}^{2}\left(t\right)\lambda_{3}^{2}\left(t\right)m_{3}.$$

From this expression, we can obtain that

$$\frac{d}{dt}Q\left(\Lambda_{t}\left(ℰ\right)\right)\leq 0,\;\forall ℰ\;\;\Leftrightarrow \;\;\frac{d}{dt}\lambda_{i}\left(t\right)\lambda_{j}\left(t\right)\leq 0,\;i\neq j,$$

which is equivalent to $\gamma_{1}\left(t\right)\;+\;\gamma_{2}\left(t\right)\;+\;\gamma_{3}\left(t\right)\;+\;\gamma_{j}\left(t\right)\geq 0$ for all j = 1,2,3.

Recall that it has been verified that the random unitary dynamics is Markovian by the definition of Equation (6) if and only if $\gamma_{i}\left(t\right)\;+\;\gamma_{j}\left(t\right)\geq 0$ for all i≠j,i,j = 1,2,3 [47], which is consistent with the result revealed by the measures based on the quantum trace distance (BLP-Markovianity) [48]. From the above, we get that Markovianity implies the completely dequantumness, while the inverse is not always true.

In summary, for several significant quantum channels, we have derived the conditions for the dynamics to be completely dequantumness, and compare them with the Markovian conditions. Their relationships are illustrated in Table 1.

From the table, we find that the completely dequantumness channel is related with Markovian channel, while they are different. There exists the Markovian channel, which induces quantumness for some ensembles. Meanwhile, there are also some completely dequantumness channel that are non-Markovian.

## 5. Conclusions

In this work, we mainly investigate the dynamics of quantumness in ensembles, and propose quantumness power and dequantumness power to characterize quantum channels. Once the channel reduces quantumness for all the ensembles at all times, we call it the completely dequantumness channel, whose relationship with the Markovian channel is studied through several examples. This work illustrates new properties of quantum channels from the perspective of the information flow in terms of quantumness brought by the interaction between the system and environment. It is worth mentioning that although we only focus on the qubit case and one special quantumness measure, the results can be generalized to arbitrary dimensions and other measures of quantumness.

There are still some problems to be further investigated. (1) From Ref. [49], we can obtain that the commutativity-preserving channels cannot increase the quantumness of ensembles, which means the quantumness power is zero for the unital qubit channel. Can we find any non-unital qubit channel without quantumness power? (2) Whether the convex combination of completely dequantumness channels is still completely dequantumness? Suppose Λ and Φ are two completely dequantumness channels, we need to check whether αΛ + (1 − α)Φ is a completely dequantumness channel or not. Since quantumness of ensembles plays an important role in quantum communication and quantum cryptography, this work is expected to be helpful in guiding quantum information tasks.

## Figures and Tables

**Table 1:** Relationship between completely dequantumness (CDQ) and Markovianity.

Channel	Completely Dequantumness	Markovianity	Relationship
Phase Damping	$\gamma_{t}\geq 0$	$\gamma_{t}\geq 0$	CDQ ⇔Markovianity
Amplitude Damping	$|G_{t}|\leq \frac{1}{4}$ and $\frac{d|G_{t}|}{dt}\leq 0$	$\frac{d|G_{t}|}{dt}\leq 0$	CDQ ${}_{⇍}^{\Rightarrow }$ Markovianity
Random Unitary	$\gamma_{1}\left(t\right)\;+\;\gamma_{2}\left(t\right)\;+\;\gamma_{3}\left(t\right)\;+\;\gamma_{j}\left(t\right)\geq 0$	$\gamma_{i}\left(t\right)\;+\;\gamma_{j}\left(t\right)\geq 0,\;i\neq j$	CDQ ${}_{⇐}^{⇏}$ Markovianity

## Appendix A

Here we give the proof of Equation (5). From the definition of the modified quantumness power, we only need to focus on the ensembles without quantumness. By Equation (2) we know that for an ensemble $ℰ\;=\;\{\left(p,\rho_{1}\right),\left(1\;-\;p,\rho_{2}\right)\}$ such that Q(ℰ) = 0, it holds that

$$r_{1}\left(\mathrm{or}\;r_{2}\right)\;=\;0\;\mathrm{or}\;n_{1}\;=\;n_{2}\;=\;n_{3}\;=\;0.$$

Towards these two cases, we calculate the quantumness of the ensemble ℰ.

(i) For the case that $r_{i}\;=\;0,i\;=\;1$ or 2, without loss of generality, we assume $r_{1}\;=\;0$, then from Equation (4) it follows that

$$Q\left(\Lambda \left(ℰ\right)\right)\;=\;p\left(1\;-\;p\right)\lambda^{2}\left(1\;-\;\lambda \right)r_{2}^{2}\sin^{2}\theta_{2}\leq \frac{1}{4}\lambda^{2}\left(1\;-\;\lambda \right),$$

and the upper bound can be achieved by the ensemble ${ℰ}_{0}\;=\;\left\{\left(\frac{1}{2},\rho_{1}\right),\left(\frac{1}{2},\rho_{2}\right)\right\}$ with $\rho_{1}\;=\;\frac{1}{2},$ and $\rho_{2}\;=\;\frac{1}{2}\left(\begin{array}{cc} 1 & e^{\;-\;i\phi }\\ e^{i\phi } & 1 \end{array}\right)$, ∀ϕ∈[0,2π).

(ii) For the case that $n_{1}\;=\;n_{2}\;=\;n_{3}\;=\;0$, from Equation (4) it follows that

$$\begin{array}{cc} Q(\Lambda (ℰ))\;= & p\left(1\;-\;p\right)\lambda^{2}\left(1\;-\;\lambda \right)\left(r_{1}^{2}\sin^{2}\theta_{1}\;+\;r_{2}^{2}\sin^{2}\theta_{2}\;-\;2r_{1}r_{2}\sin\theta_{1}\sin\theta_{2}\cos\left(\phi_{1}\;-\;\phi_{2}\right)\right)\\ \leq & p\left(1\;-\;p\right)\lambda^{2}\left(1\;-\;\lambda \right)(r_{1}^{2}\sin^{2}\theta_{1}\;+\;r_{2}^{2}\sin^{2}\theta_{2}\;+\;2r_{1}r_{2}|\sin\theta_{1}\sin\theta_{2}\left|\right)\\ \;= & p\left(1\;-\;p\right)\lambda^{2}\left(1\;-\;\lambda \right)(r_{1}|\sin\theta_{1}|\;+\;r_{2}|\sin\theta_{2}{\left|\right)}^{2}\leq \lambda^{2}\left(1\;-\;\lambda \right), \end{array}$$

and the upper bound can be achieved by the ensemble ${ℰ}_{0}\;=\;\left\{\left(\frac{1}{2},\rho_{1}\right),\left(\frac{1}{2},\rho_{2}\right)\right\}$ with $\rho_{1}\;=\;\frac{1}{2}\left(\begin{array}{cc} 1 & e^{\;-\;i\phi_{1}}\\ e^{i\phi_{1}} & 1 \end{array}\right)$ and $\rho_{2}\;=\;\frac{1}{2}\left(\begin{array}{cc} 1 & \;-\;e^{\;-\;i\phi_{2}}\\ e^{i\phi_{2}} & 1 \end{array}\right)$, $\forall \phi_{i}\in \left[0,2\pi \right),i\;=\;1,2$.

Combining these two cases, we get that the modified quantumness power of the amplitude damping channel Λ is

$$C_{2}^{{}^{\prime }}\left(\Lambda \right)\;=\;\max_{\left\{{ℰ}_{2}:Q\left({ℰ}_{2}\right)\;=\;0\right\}}Q\left(\Lambda \left({ℰ}_{2}\right)\right)\;=\;\lambda^{2}\left(1\;-\;\lambda \right).$$
